# Improved Bald Eagle Search Optimization with Synergic Deep Learning-Based Classification on Breast Cancer Imaging

**DOI:** 10.3390/cancers14246159

**Published:** 2022-12-14

**Authors:** Manar Ahmed Hamza, Hanan Abdullah Mengash, Mohamed K Nour, Naif Alasmari, Amira Sayed A. Aziz, Gouse Pasha Mohammed, Abu Sarwar Zamani, Amgad Atta Abdelmageed

**Affiliations:** 1 Department of Computer and Self Development, Preparatory Year Deanship, Prince Sattam bin Abdulaziz University, AlKharj 16242, Saudi Arabia; 2 Department of Information Systems, College of Computer and Information Sciences, Princess Nourah bint Abdulrahman University, P.O. Box 84428, Riyadh 11671, Saudi Arabia; 3 Department of Computer Sciences, College of Computing and Information System, Umm Al-Qura University, Makkah 24211, Saudi Arabia; 4 Department of Information Systems, College of Science & Art at Mahayil, King Khalid University, Muhayil 63311, Saudi Arabia; 5 Department of Digital Media, Faculty of Computers and Information Technology, Future University in Egypt, New Cairo 11835, Egypt

**Keywords:** computer-aided diagnosis, medical imaging, breast cancer, histopathological images, deep learning, bald eagle search algorithm

## Abstract

**Simple Summary:**

The manual process of microscopic inspections is a laborious task, and the results might be misleading as a result of human error occurring. This article presents a model of an improved bald eagle search optimization with a synergic deep learning mechanism for breast cancer diagnoses using histopathological images (IBESSDL-BCHI). The performance validation of the IBESSDL-BCHI system was tested utilizing the benchmark dataset, and the results demonstrate that the IBESSDL-BCHI model has shown better general efficiency for BC classification.

**Abstract:**

Medical imaging has attracted growing interest in the field of healthcare regarding breast cancer (BC). Globally, BC is a major cause of mortality amongst women. Now, the examination of histopathology images is the medical gold standard for cancer diagnoses. However, the manual process of microscopic inspections is a laborious task, and the results might be misleading as a result of human error occurring. Thus, the computer-aided diagnoses (CAD) system can be utilized for accurately detecting cancer within essential time constraints, as earlier diagnosis is the key to curing cancer. The classification and diagnosis of BC utilizing the deep learning algorithm has gained considerable attention. This article presents a model of an improved bald eagle search optimization with a synergic deep learning mechanism for breast cancer diagnoses using histopathological images (IBESSDL-BCHI). The proposed IBESSDL-BCHI model concentrates on the identification and classification of BC using HIs. To do so, the presented IBESSDL-BCHI model follows an image preprocessing method using a median filtering (MF) technique as a preprocessing step. In addition, feature extraction using a synergic deep learning (SDL) model is carried out, and the hyperparameters related to the SDL mechanism are tuned by the use of the IBES model. Lastly, long short-term memory (LSTM) was utilized to precisely categorize the HIs into two major classes, such as benign and malignant. The performance validation of the IBESSDL-BCHI system was tested utilizing the benchmark dataset, and the results demonstrate that the IBESSDL-BCHI model has shown better general efficiency for BC classification.

## 1. Introduction

Worldwide, the number of cancer cases is increasing at a faster rate than it ever has before. Multimodal medical imaging is utilized for diagnosing distinct kinds of cancers with the help of whole slide images (WSIs), MRIs, CT scans, and more [1]. The manual detection of cancer, with the help of imaging, was a time-consuming procedure, and it relied on the expertise of the consultant or doctor [2]. As a result, a high death rate is linked with late cancer detection, and a computer-aided diagnosis (CAD) technique which recognizes a tumor precisely within the time limitations has become necessary. Therefore, initial identification was the key factor to curing cancer [3]. The golden standard for determining the breast cancer (BC) prognosis was, until now, a pathological analysis. A pathological analysis generally acquires cancer samples via excision, puncture, and so on [4]. Hematoxylin binds to deoxyribonucleic acid (DNA) to highlight the nucleus, whereas eosin binds to proteins and emphasizes other frameworks. A precise prognosis of BC needs skilled histopathologists and consumes more effort and time to finish the task. Additionally, the diagnosis outcome of discrete histopathologists were the not same, and they mainly relied on the prior knowledge of the histopathologists [5]. This lead to an average diagnosis accuracy and a lower diagnosis consistency of 75%.

However, the study of the histopathological images (HIs) is a challenging and time-consuming task which requires professional expertise. Additionally, the analysis outcome can be affected through the experience level of the diagnosticians involved [6]. Thus, the computer-aided study of HIs serves a crucial role in BC diagnosis. However, the procedure of advancing the tools to perform the study has been hindered by the following difficulties: Firstly, the HIs of BC were finely grained, higher-resolution images which represent complex textures and rich geometric structures. The changes in a class and the consistency among the classes could cause the categorization to be highly complex, particularly for situations with many classes [7,8]. Secondly, we considered the constraints of feature extraction (FE) techniques for the HIs of BC. 

Conventional FE approaches such as the gray-level co-occurrence matrix (GLCM) and scale invariant feature transform (SIFT) depend upon supervised information. In addition to that, earlier knowledge about the data was required for selecting the valuable features that cause the FE efficiency to be low and the computational load to be high [9]. Therefore, this might result in the final model generating the worst classification of outcomes. Deep learning (DL) methods are capable of extracting features automatically, restoring information from data mechanically, and studying enhanced abstract data representations [10]. It could resolve the issues of conventional FS, and it has been applied in computer vision (CV) successfully and also in biomedical science and in other domains.

This article presents a model of an improved bald eagle search optimization with a synergic deep learning mechanism for breast cancer diagnosis using histopathological images (IBESSDL-BCHI). The proposed IBESSDL-BCHI model follows image preprocessing using a median filtering (MF) technique as a preprocessing step. In addition, a feature extraction using a synergic deep learning (SDL) model was carried out, and the hyperparameters related to the SDL mechanism were tuned by the use of an IBES model. At last, the long short-term memory (LSTM) system was utilized for precisely categorizing the HIs into two major classes: benign and malignant. The performance validation of the IBESSDL-BCHI method was tested using the benchmark dataset. The key contributions of the paper are highlighted as follows:

- An intelligent IBESSDL-BCHI technique comprising of MF-based pre-processing, SDL feature extraction, IBES-based parameter optimization, and an LSTM model for BC detection and classification using HIs is presented. To the best of our knowledge, the IBESSDL-BCHI model has never been presented in the literature.
- A novel IBES algorithm is designed by the integration of oppositional-based learning with the traditional BES algorithm.
- Hyperparameter optimization of the SDL model using the IBES algorithm using cross-validation helps to boost the classification outcome of the IBESSDL-BCHI model for unseen data.

## 2. Related Works

In the study that was conducted earlier [11], a new patch-related DL technique named Pa-DBN-BC was suggested for the detection and classification of BC on histopathology images using a Deep Belief Network (DBN). In this technique, the features can be derived via conducting supervised fine-tuned and unsupervised pre-training stages. The network automatically extracts the features from the image stains. In the literature [12], the researchers compared two ML techniques for the automatic classification of BC histology images as either malevolent or benevolent and their respective sub-classes. The initial technique was designed based on the abstraction of a group of handcrafted features that are encrypted with Bag of Words (BoW) and locality-constrained linear coding, and it was well-trained through an SVM classifier. The next method was designed based on a CNN model. 

In the literature [13], the researchers suggested a method to use DL techniques with convolutional layers for the extraction of valuable visual features and classify the BC. It was revealed that such DL techniques can derive superior features in comparison with the handcrafted FS methods. It further suggests a new advanced strategy to achieve the primary objective. Further, the model can be effectively improved through the progressive merging of the DL methods with weak classifiers as a stronger classifier. Xie et al. [14] presented a new model for the analysis of HIs of BC through unsupervised and supervised deep CNN networks. At first, it adapted Inception_ResNet_V2 and Inception_V3 infrastructures to binary and multi-class problems of BC-HI classification with the help of Transfer Learning (TL) approaches.

In the study that was conducted earlier [15], the authors recommended a system for BC classification with an Inception Recurrent Residual (IRRCNN) method. The proposed IRRCNN is a powerful DCNN method since it combines the robustness of Recurrent RCNN, v4ResNet, and the Inception technique. The proposed IRRCNN method achieved better outcomes towards the equivalent networks, Inception Networks, and the RCNNs in terms of an object recognition task. Yang et al. [16] suggested to employ further regional-level supervision for BC classification of the HIs using the CNN technique. In this method, the RoIs were localized and utilized for guiding the interest of the classifier network concurrently. The presented supervised attention algorithm precisely stimulated the neurons in the diagnostic-related areas, whereas it suppressed the stimulations in the inappropriate and noisy regions. 

Ali et al. [17] presented an effective DL model to exploit the small dataset and learn generalizable and domain-invariant representation in various medical imaging applications for diseases such as malaria, Diabetic Retinopathy, and tuberculosis. This model was named the Incremental Modular Network Synthesis (IMNS), and the resultant CNNs were the Incremental Modular Networks (IMNets). The authors in the study conducted earlier [18] developed a cloud-enabled Android app to detect breast cancer using the ResNet101 model. The proposed framework was cost-effective, and it demanded less human intervention as it was cloud integrated. So, a lower performance load was placed on the edge devices. Narayanan et al. [19] presented a novel Deep Convolutional Neural Network architecture for the Invasive Ductal Carcinoma (IDC) classification process.

## 3. The Proposed Model

**Figure 1:**
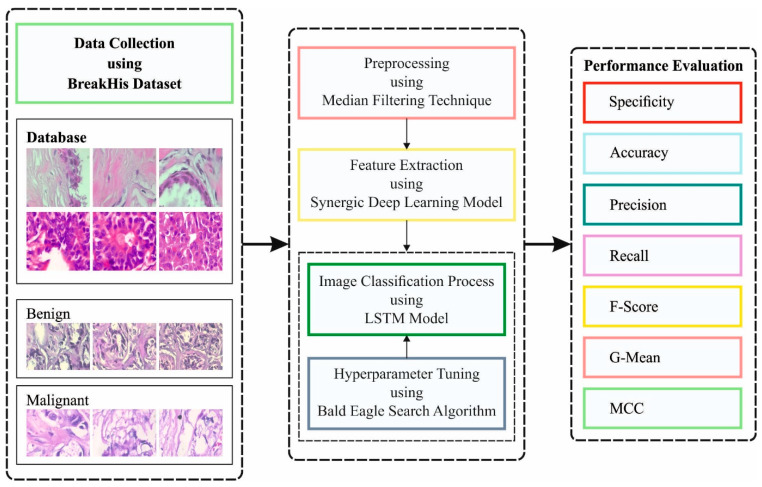
Workflow of the proposed IBESSDL-BCHI methodology.

In the current study, a new IBESSDL-BCHI method has been developed for the recognition and classification of BC using the HIs. The presented IBESSDL-BCHI method follows a series of processes, namely, MF-based noise removal, SDL feature extraction, IBES-based hyperparameter optimization, and LSTM classification. The design of the IBES algorithm helps in precisely categorizing the HIs into two major classes, namely, benign and malignant. Figure 1 depicts the workflow of the proposed IBESSDL-BCHI approach.

### 3.1. Image Preprocessing

Initially, the Median Filtering (MF) technique was utilized to preprocess the input HIs. MF is a nonlinear digital filter method that is frequently utilized in the removal of noise from images/signals. Such noise reduction is a classical pre-processing phase that is performed to enhance the outcomes in the later processes. The MF approach smoothens the HIs [20], and its steps are as follows: 

Step1: The 3 × 3 kernel needs zero padding 3/2 = 1 column of 0′s at the left as well as the right edges, but it needs 3/2 = 1 row of 0′s at the upper as well as the bottom edges.

Step 2: To process the primary component, this approach covers 3 × 3 kernels with the center of them pointing at the initially handled component. The data, arranged in the kernel, were recorded with respect to the value, and the attained median value is obtained.

Step 3: We repeated the process for all of the elements until the final value was obtained.

The MF function calculates the median of every pixel in the kernel window, and the central pixel is interchanged with this median value. It can be extremely effectual in the extraction of salt-and-pepper noises. Notably, during the application of the Gaussian and box filters, the filter values to the central element remain a value that cannot occur in the original images. However, this is not the case in the MF approach since the central element is continuously exchanged with any of the pixel values of the images. This phenomenon decreases the noise in an efficient manner. The size of the kernel is a positive odd integer, and the median function is calculated as given in Equation (1).

(1) $$Med\left(X\right)=\left\{\begin{array}{c} X\left[\frac{n}{2}\right]\\ \frac{\left(\frac{X\left[n-1\right]}{2}+\frac{X\left[n+1\right]}{2}\right)}{2} \end{array}\right.$$

Here, X refers to the orderly list of values from the dataset and n signifies the amount of values from the dataset.

### 3.2. SDL-Based Feature Extraction

After the image preprocessing, the SDL model was utilized to derive the feature vectors. During the feature extraction procedure, the pre-processed images were fed into the SDL module to obtain a beneficial set of feature vectors [21].

**Figure 2:**
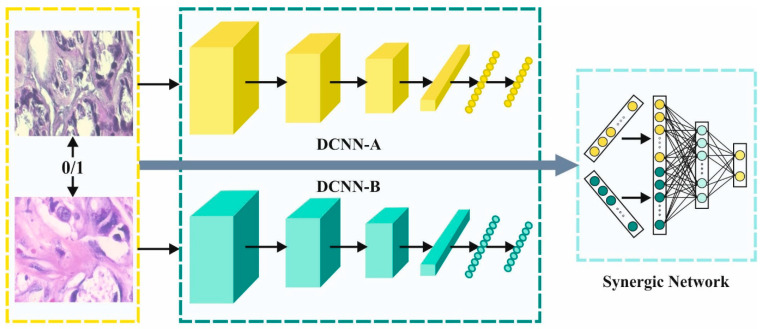
Architecture of SDL network.

The SDL model extracts the feature subsets from the pre-processed images. It represents the $SDL^{k}$ through three main elements such as k DCNN component, the input layer and the $C_{k}^{2}$ synergic network (SN). Every DCNN component of the network provides an independent learning representation in the input dataset. The SN consists of the FC architecture to ensure that the input layers belong to the same class, and it offers remedial comments. Afterward, the SDL system is classified into three sub-models. Figure 2 illustrates the architecture of the SDL network.

#### 3.2.1. Components of DCNN

Due to the implicit nature of ResNet, ResNet-50 was exploited for initializing every DCNN component $\left(a=1,2,\;\ldots ,\;n\right)$. Therefore, it can be indicated that the DCNN network comprises of VGGNet, AlexNet, and GoogLeNet which correspond to the SDL method. This module was trained using the data sequence $X=\left\{x^{\left(1\right)},\;x^{\left(2\right)},\;\ldots \;,\;x^{\left(M\right)}\right\}$ and a series of the last class label, $Y=\left\{y^{\left(1\right)},\;y^{\left(2\right)},\;\ldots \;,\;y^{\left(M\right)}\right\}$. The aim is to progress with a group of variables θ which make sure that the CE loss is offered as follows:

(2) $$log\left(\theta \right)=-\frac{1}{M}\left[\overset{M}{\underset{a=1}{\sum }}\overset{K}{\underset{b=1}{\sum }}1\left\{y^{\left(1\right)}=b\right\}log\frac{e^{Z_{b}^{\left(a\right)}}}{\sum_{l=1}^{K}z_{l}^{\left(a\right)}}\right]$$

In Equation (2), n represents the class number and $Z^{\left(a\right)}=F\left(x^{\left(a\right)},\;\theta \right)$ denotes the forward computation. The group of variables obtained for DCNN-a indicates that $\theta^{a}$ and the variable do not assign any massive DCNN units.

#### 3.2.2. SDL Model

The DCNN component, using the synergic labels of the pair of embedded and the input layers, is exploited for FC learning. Assuming that $\left(Z_{A},\;Z_{B}\right)$ are a data pair given as the input for two DCNN features (DCNNa, DCNNb) as follows,

(3) $$f_{A}=F\left(Z_{A},\;\theta^{\left(a\right)}\right)$$

(4) $$f_{B}=F\left(Z_{B},\;\theta^{\left(a\right)}\right)$$

Next, the deep feature from the whole dataset is embedded as $f_{A^{\circ }B}$, and the outcomes using the synergic label are given below.

(5) $$ys\left(Z_{A},\;Z_{B}\right)=\left\{\begin{array}{l} 1\;if\;y_{A}=y_{B}\\ 0\;if\;YA\neq y_{B} \end{array}\right.$$

To resolve the shortcoming, the percentage data pair from the class need to be higher. So, a simple-to-zero value is used to gauge the synergic signals using an alternate sigmoid layer, and the binary CE loss is as follows.

(6) $$l^{S}\left(\theta^{S}\right)=y_{S}log{\hat{y}}_{s}+\left(1-y_{S}\right)log\left(1-{\hat{y}}_{s}\right)$$

In Equation (6), $\theta^{S}$ denotes the SN attribute and ${\hat{y}}_{s}$ indicates the SN forward computation. This validates that the input dataset pair belong to the same class, and it offers the option to remedy the synergic error.

#### 3.2.3. Training and Testing Processes

Once the training is completed, the features of both the DCNN component and the SN become improved.

(7) $$\left\{\begin{array}{l} \theta^{\left(a\right)}\left(z+1\right)=\theta^{\left(a\right)}\left(z\right)-\eta \left(z\right).{△}^{\left(a\right)}\\ \theta^{S\left(a\right)}\left(z+1\right)=\theta^{S\left(a\right)}\left(z\right)-\eta \left(z\right).{△}^{S\left(a,b\right)} \end{array}\right.$$

In Equation (7), $\eta \left(z\right)$ and $S\left(a,\;b\right)$ indicate the learning rate and SN between DCNNa and DCNNb, respectively, as given below.

(8) $${△}^{\left(a\right)}=\frac{\partial l^{\left(a\right)}\left(\theta^{\left(a\right)}\right)}{\partial \theta^{\left(a\right)}}+\lambda \overset{n}{\underset{b=1,b\neq a}{\sum }}\frac{\partial l^{S\left(a\right)}\left(\theta^{S\left(a,b\right)}\right)}{\partial \theta^{S\left(a,b\right)}}$$

(9) $${△}^{S\left(a\right)}=\frac{\partial l^{S\left(a\right)}\left(\theta^{S\left(a,b\right)}\right)}{\partial \theta^{S\left(a,b\right)}}$$

Here, λ denotes the trade-off between the sub-model of the classifiers and the synergic errors. The relationship between the trained process of the $SDL^{2}$ models increases. In the trained $SDL^{k}$, the testing dataset x is classified using the DCNN unit, while it provides the prediction vector $P^{\left(a\right)}=$ $\left(p_{1}^{\left(a\right)},\;p_{2}^{\left(a\right)},\;\ldots ,\;p_{k}^{\left(a\right)}\right)$ which is activated from the resulting FC layer. The class labels of the testing dataset are evaluated as follows.

(10) $$y\left(Z\right)=\underset{v}{\mathrm{argmax}}\{\overset{k}{\underset{u=1}{\sum }}p_{1}^{\left(u\right)},\ldots ,\overset{k}{\underset{u=1}{\sum }}p_{v}^{\left(u\right)},\ldots ,\overset{k}{\underset{u=1}{\sum }}p_{K}^{\left(u\right)}$$

### 3.3. Hyperparameter Tuning Using IBES Algorithm

In this study, the hyperparameters related to the SDL mechanism are fine-tuned with the help of the IBES model. BES is a meta-heuristic optimization approach that imitates the behavior of bald eagle hunting [22]. This procedure has three phases, namely, selecting the space, searching in the space, and swooping. Initially, the bald eagles choose the best place in terms of the food amount. Next, the eagle searches for prey within the nominated place. In the optimally attained location in the previous stage, the eagle swoops to determine the optimal hunting site, which is the last phase.

a. Selection space: In this phase, a novel position is produced based on the subsequent formula.

(11) $$P_{new}\left(i\right)=P_{best}+\alpha .r.\left(P_{mean}-P\left(i\right)\right)$$

In Equation (11), $P_{new}\left(i\right)$ denotes the i-th recently produced location, $P_{best}$ refers to the optimally attained location, $P_{mean}$ indicates the mean location, α represents a control gain [1.5, 2], and r indicates an arbitrary integer that lies in the range of [0, 1]. The fitness of every novel location is estimated; if the novel location $\left(P_{new}\right)$ offers a better fitness than the offered one $P_{best}$, then the novel location is allocated by $P_{best}.$

b. Searching in space: After the allocation of the optimal search space $\left(P_{best}\right)$ is completed, the process upgrades the location of the eagles within the searching space. The update module is given herein.

(12) $$P_{new}\left(i\right)=P\left(i\right)+y\left(i\right).\left(P\left(i\right)-P\left(i+1\right)\right)+x\left(i\right).\left(P\left(i\right)-P_{mean}\right)$$

In Equation (12), $P_{new}\left(i\right)$ denotes the i−th recently produced position, $P_{mean}$ indicates the mean location, and x and y denote the directional coordinates for the i−th  location as given below.

(13) $$x\left(i\right)=\frac{xr\left(i\right)}{\;\max\left(\left|xr\right|\right)};xr\left(i\right)=r\left(i\right).sin\left(\theta \left(i\right)\right)\;y\left(i\right)=\frac{yr\left(i\right)}{\max\left(\left|yr\right|\right)};yr\left(i\right)=r\left(i\right).cos\left(\theta \left(i\right)\right)$$

$$\theta \left(i\right)=a.\pi .rand;r\left(i\right)=\theta \left(i\right).R.rand$$

In Equation (13), a indicates a control variable that is utilized to determine the corner between the searching point and the central point, and it takes the values in the range of [5, 10]. R denotes a variable within [0.5, 2], and it is utilized to determine the number of searching cycles. The fitness of the novel position is estimated, and the $P_{best}$ values are upgraded based on the attained outcomes.

c. Swooping: In this phase, the eagle moves towards the prey from the optimally attained location. The hunting model is given in the following expression.

(14) $$P_{new}\left(i\right)=rand.P_{best}+x1\left(i\right).\left(P\left(i\right)-c_{1}.P_{mean}\right)+y1\left(i\right).\left(P\left(i\right)-c_{2}.P_{best}\right)\;$$

In Equation (14), $c_{1}$ and $c_{2}$ denote two arbitrary integers that lie in the range of [1,2]; x1 and y1 indicate the directional coordinates that are determined as follows.

(15) $$x1\left(i\right)=\frac{xr\left(i\right)}{\;\max\left(\left|xr\right|\right)};xr\left(i\right)=r\left(i\right).sinh\left(\theta \left(i\right)\right)\;y1\left(i\right)=\frac{yr\left(i\right)}{\max\left(\left|yr\right|\right)};yr\left(i\right)=r\left(i\right).cosh\left(\theta \left(i\right)\right)$$

$$\theta \left(i\right)=a.\pi .rand;r\left(i\right)=\theta \left(i\right)$$

Here, Npop denotes the number of locations (population size), and MaxIter indicates the max  number of iterations.

The IBES model is derived by the inclusion of the Oppositional-Based Learning (OBL) concept to optimize the efficiency of BES. The OBL model was highlighted by Tizoosh et al. to estimate the individual fitness, and it relates to their equivalent opposite number after bringing the optimum one into the next iteration in the OBL approach, and it is determined as follows. 

Opposite number: We assume that x is a real number and $x\in \left[lb,\;ub\right],$ the next the opposite number $\bar{x}$, is provided by the subsequent value as shown in Equation (16).

(16) $$\bar{x}=ub+lb-x$$

Here, lb and ub correspondingly denote the lower and upper boundaries, respectively.

Opposite vector: When $x=\left(x_{1},x_{2},\;\ldots x_{D}\right)$, $x_{1},x_{2},\ldots x_{D}$ denote the real numbers and $x\in \left[lb,\;ub\right]$, and then ${\bar{x}}_{i}$ is computed as given below.

(17) $${\bar{x}}_{i}=lb_{i}+ub_{i}-x_{i}.$$

At last, the current solution is located by ${\bar{x}}_{i}$, if $f\left(\bar{x}\right)<f\left(x\right)$

The IBES method resolves the Fitness Function (FF) to obtain a superior classification performance. In this study, a reduced classifier error rate is treated as FF as given below.

(18) $$fitness\left(x_{i}\right)=ClassifierErrorRate\left(x_{i}\right)\;=\frac{number\;of\;misclassified\;samples}{Total\;number\;of\;samples}*100$$

### 3.4. LSTM-Based Classification

During the image classification process, the LSTM model is used to precisely categorize the HIs under two major classes, namely, benign and malignant. Being a variant of the RNN model, the LSTM model basically differs from the classical ANN [23]. Both the LSTM and RNN are sequence-based methods with internal self-looped repeating networks. These determines the temporal relationship amid the sequential datasets and preserve the previous information.

In the current study, the repeated module has a simple framework (Tanh layer). $f_{t}$ denotes the output of the forget gate a, for which the values lie in the range of [0, 1].

For the above explanation, the mathematical expression is given below.

(19) $$f_{f}=\sigma \left(W_{f}\cdot \left[h_{t-1},x_{t}\right]+b_{f}\right)$$

The next layer of the LSTM blocks are named as an ‘input gate’ layer as shown below.

(20) $$i_{t}=\sigma \left(W_{i}\cdot \left[h_{t-1},x_{t}\right]+b_{i}\right)\;$$

(21) $$\tilde{C}=\phi \left(W_{C}\cdot \left[h_{t-1},x_{\zeta }\right]+b_{C}\right)\;$$

Afterwards, the older cell state $C_{t-1}$ should be upgraded to the cell state, $C_{t}$. The output of the forget gate $f_{t}$ is the decision to forget, and $i_{r}$ defines that a novel cell state has been added, i.e., ${\tilde{C}}_{t}$. The update procedure of $C_{t}$ is described below.

(22) $$C_{t}=f_{t^{*}}C_{t-1}+i_{t}*{\tilde{C}}_{t}\;$$

At last, the interacting layer is named the ‘output gate’ layer. The procedure of producing an output of the LSTM block is demonstrated herein.

(23) $$0_{t}=\sigma {\left(W_{0}*\left[h_{t-1},x_{t}\right]+b_{0}\right)}^{*}\phi \left(C_{t}\right)\;$$

In Equation (23), 0 shows the activation function, namely, Sigmoid, and ϕ refers to the Tanh function. Given that $\theta =\left\{W,\;b\right\}$ characterizes the variable vector of the network, $W=[W_{f}W_{i},W_{c},W_{o}\;|$ and $b=\left[b_{f},\;b_{i},\;b_{C},\;b_{o}\right]$ indicate the weight and bias, respectively. The forward formulation in Equations (20)–(23) is indicated by $=NN\left(X;\theta \right)$:

(24) $$L\left(\theta \right)LSTM=\frac{J}{N}\overset{N}{\underset{i=1}{\sum }}|NN\left(x_{i;}\theta \right)-y_{i}{|}^{2}$$

In Equation (24), N indicates the overall number of labeled datasets. In the training course of LSTM, θ is tuned continuously by diminishing the loss function via an optimized technique, namely, SGD.

## 4. Results and Discussion

**Figure 3:**
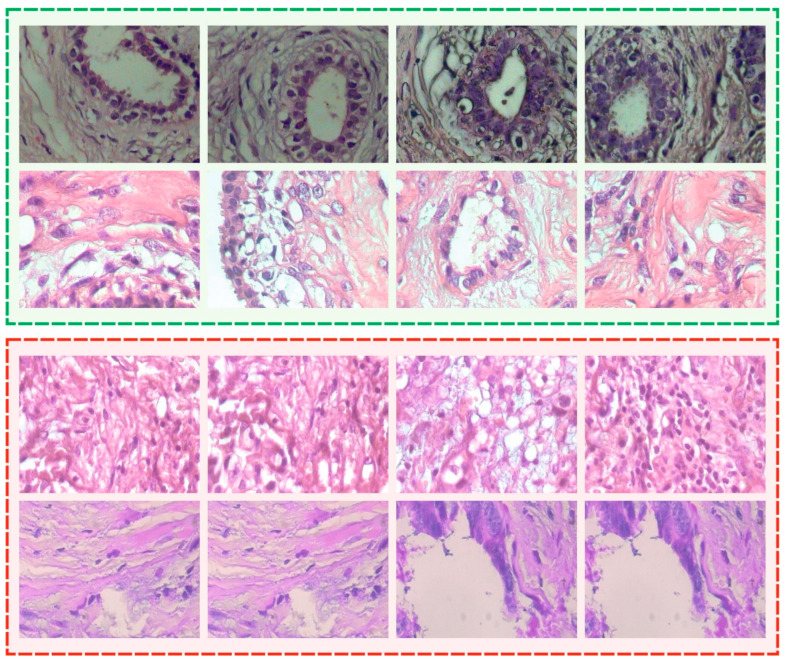
Sample images.

The proposed IBESSDL-BCHI method was experimentally validated using a benchmark Breast Cancer Histopathological Database (BreakHis) dataset [4] comprising 1820 HIs. The dataset holds a total of 588 images under the benign class and 1,232 images under the malignant class, and the details are given in Table 1. A few sample images are showcased in Figure 3.

**Figure 4:**
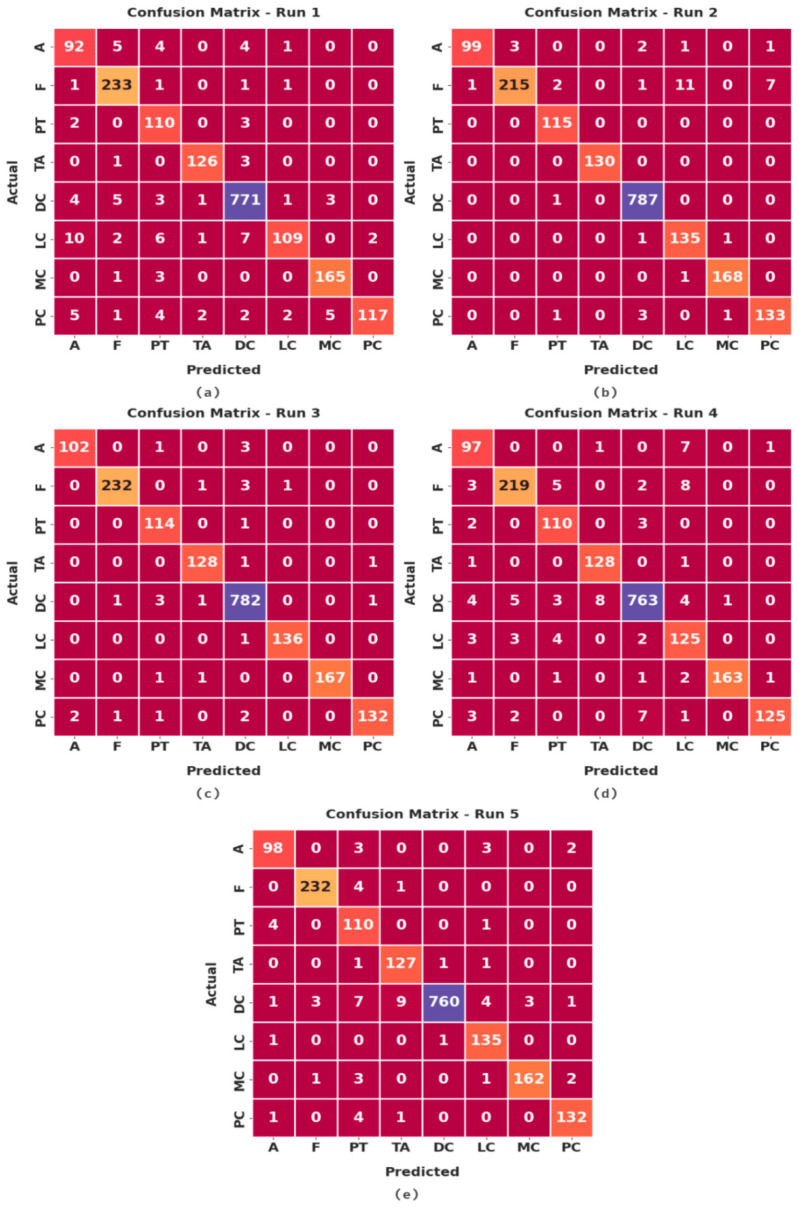
Confusion matrices of the proposed IBESSDL-BCHI approach; (**a**) Run 1, (**b**) Run 2, (**c**) Run 3, (**d**) Run 4, and (**e**) Run 5.

Figure 4 illustrates a set of confusion matrices generated by the proposed IBESSDL-BCHI method on the test dataset. In run 1, the IBESSDL-BCHI model classified 92 images under class ‘A’, 233 images under class ‘F’, 110 images under class ‘PT’, 126 images under ‘TA’, 771 images under ‘DC’, 109 images under class ‘LC’, 165 images under ‘MC’, and 117 images under ‘PC’. 

**Figure 5:**
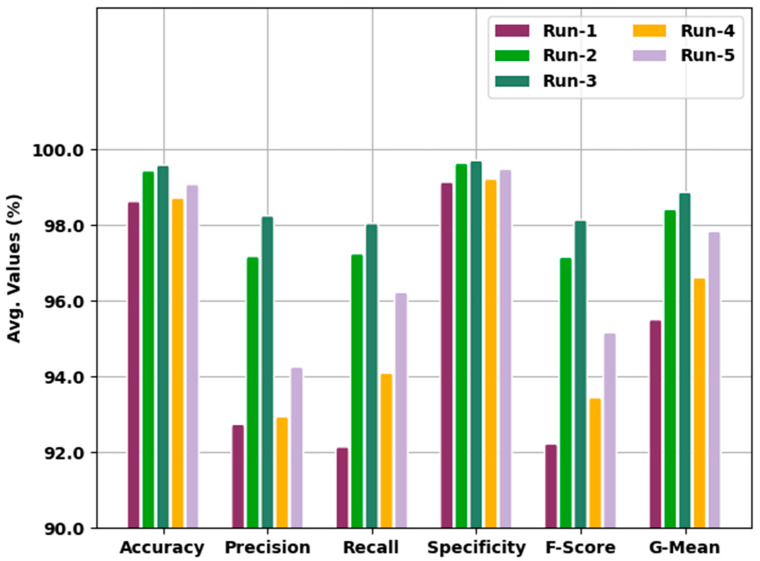
Analytical results of the IBESSDL-BCHI approach during distinct runs.

Table 2 and Figure 5 show the analytical outcomes of the IBESSDL-BCHI model during distinct test runs in terms of its accuracy ($accu_{y}$), precision ($prec_{n}$), recall ($reca_{l})$, specificity ($spec_{y}$), F-score ($F_{score}$), and G-mean ($G_{mean}$). The experimental values infer that the proposed IBESSDL-BCHI method attained the maximum number of classification results under every run. For example, in run 1, the IBESSDL-BCHI technique attained the average $accu_{y}$, $prec_{n}$, $reca_{l}$, $spec_{y}$, $F_{score}$, and $G_{mean}$ values which were 98.67%, 92.79%, 92.19%, 99.18%, 92.27%, and 95.55%, respectively. Additionally, in run 2, the proposed IBESSDL-BCHI approach reached the average $accu_{y}$, $prec_{n}$, $reca_{l}$, $spec_{y}$, $F_{score}$ and $G_{mean}$ values which were 99.48%, 97.22%, 97.29%, 99.68%, 97.20% and 98.46% correspondingly. In addition to these, in run 4, the IBESSDL-BCHI model accomplished the average $accu_{y}$, $prec_{n}$, $reca_{l}$, $spec_{y}$, $F_{score}$, and $G_{mean}$ values which were 98.76%, 92.99%, 94.14%, 99.26%, 93.49%, and 96.66% correspondingly. Along with that, in run 5, the IBESSDL-BCHI methodology achieved the average $accu_{y}$, $prec_{n}$, $reca_{l}$, $spec_{y}$, $F_{score}$, and $G_{mean}$ values which were 99.12%, 94.30%, 96.27%, 99.52%, 95.21% and 97.88% correspondingly.

**Figure 6:**
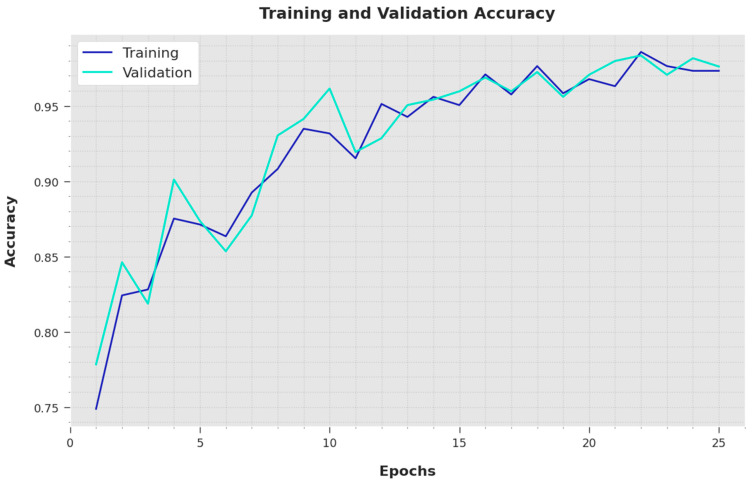
TA and VA analyses results of the IBESSDL-BCHI approach.

Both the Training Accuracy (TA) and Validation Accuracy (VA) values obtained using the proposed IBESSDL-BCHI method using the test dataset are depicted in Figure 6. The outcomes demonstrate that the proposed IBESSDL-BCHI methodology achieved the highest TA and VA values, while the VA values were superior to the TA values.

**Figure 7:**
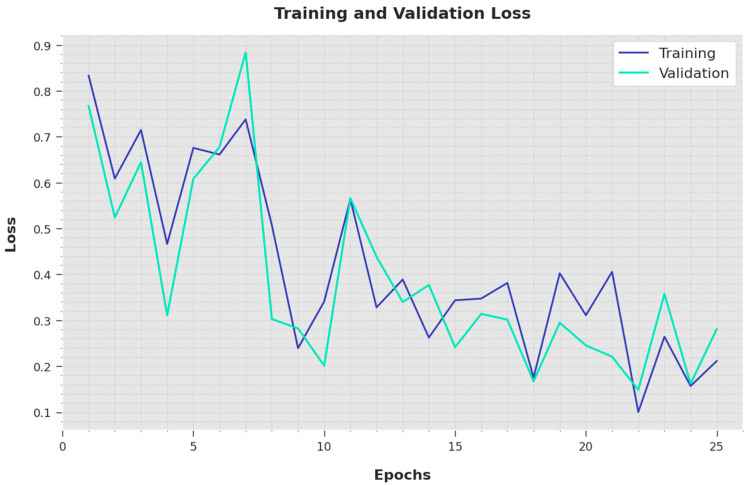
TL and VL analyses results of the IBESSDL-BCHI methodology.

Both the Training Loss (TL) and Validation Loss (VL) values attained by the proposed IBESSDL-BCHI methodology using the test data are depicted in Figure 7. The outcomes illustrate that the proposed IBESSDL-BCHI technique demonstrated minimal TL and VL values, while the VL values seemed to be smaller than the TL values. 

**Figure 8:**
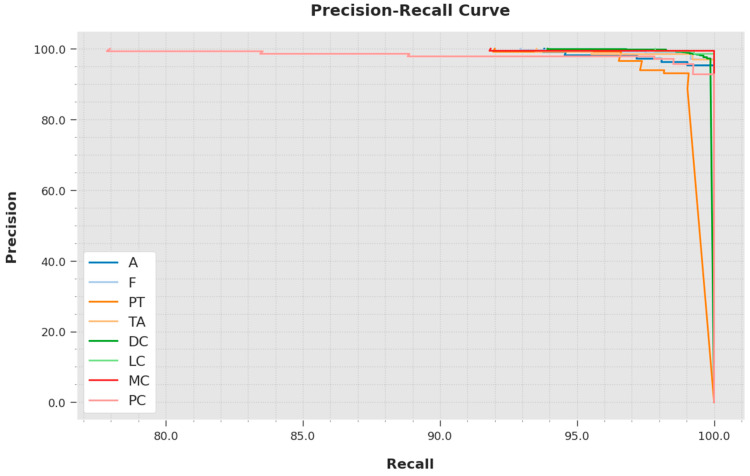
Precision-recall outcomes of the IBESSDL-BCHI approach.

A brief precision-recall inspection was conducted with the IBESSDL-BCHI method using the test data, and the results are depicted in Figure 8. It is to be noted that the proposed IBESSDL-BCHI approach obtained the maximal precision-recall performance under all of the classes.

**Figure 9:**
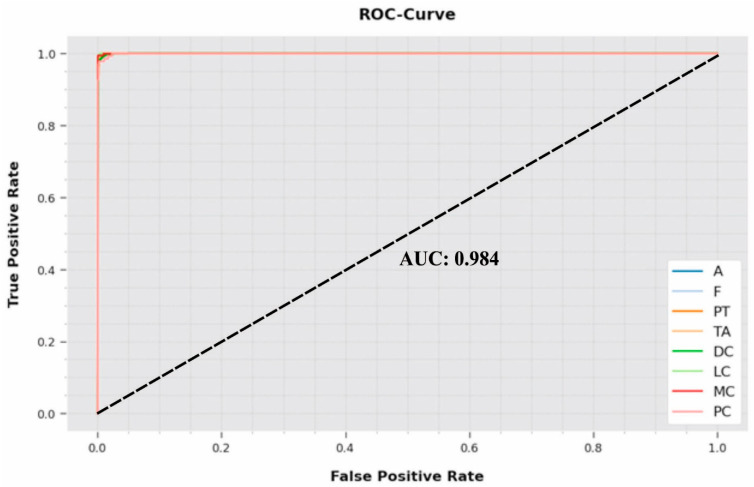
ROC curve analysis results of the IBESSDL-BCHI approach.

A comprehensive ROC inspection was conducted on the proposed IBESSDL-BCHI system using the test dataset, and the results are portrayed in Figure 9. The outcomes show that the proposed IBESSDL-BCHI method depicted capability in categorizing the test dataset into dissimilar classes.

**Figure 10:**
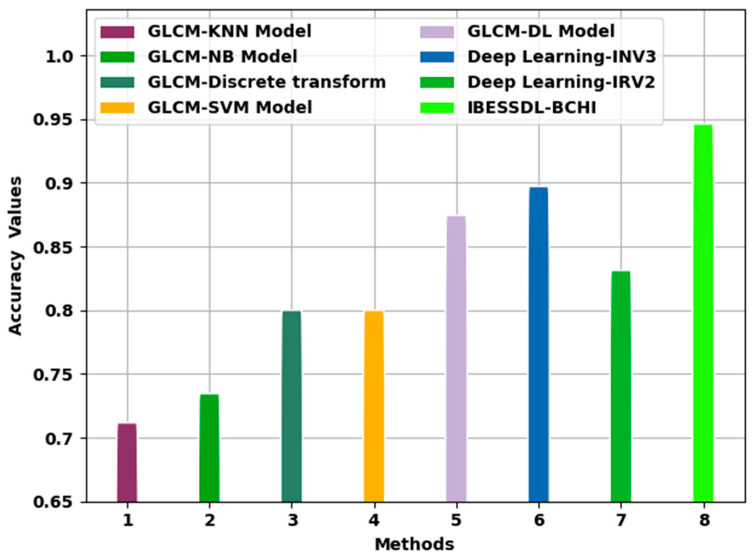
$Accu_{y}$ analysis results of the IBESSDL-BCHI approach and other existing methodologies.

Table 3 provides the overall comparison analysis outcomes achieved by the proposed IBESSDL-BCHI method and other existing models [14,24]. Figure 10 portrays the comparative examination outcomes of the IBESSDL-BCHI technique and other techniques in terms of $accu_{y}$. The figure implies that the proposed IBESSDL-BCHI system achieved enhanced $accu_{y}$ values. With respect to $accu_{y}$, the IBESSDL-BCHI approach obtained a maximum $accu_{y}$ of 0.9963, whereas the rest of the methods such as the GLCM-KNN, GLCM-NB, GLCM-Discrete transform, GLCM-SVM, GLCM-DL, DL-INV3, and DL-INV2 models attained low $accu_{y}$ values which were 0.7617, 0.7845, 0.8500, 0.8500, 0.9244, 0.9471, and 0.8812, respectively.

**Figure 11:**
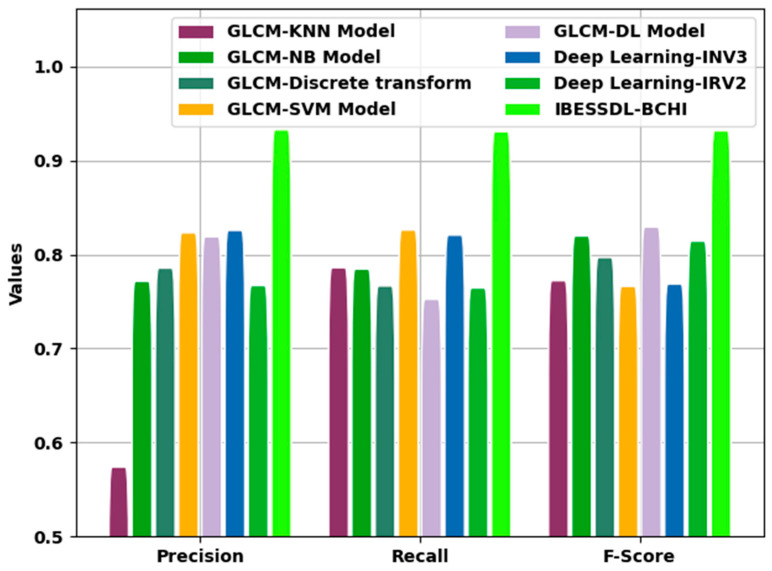
Comparative analysis outcomes of the proposed IBESSDL-BCHI approach and other existing methodologies.

Figure 11 demonstrates the comparative investigation outcomes attained by the proposed IBESSDL-BCHI approach and other techniques in terms of $Prec_{n}$, $reca_{l}$, and $F_{score}$. The figure reveals that the proposed IBESSDL-BCHI methodology produced maximum $Prec_{n}$, $reca_{l}$, and $F_{score}$ values. With respect to $prec_{n}$, the IBESSDL-BCHI method obtained a superior $prec_{n}$ value of 0.9829, whereas the other models such as the GLCM-KNN, GLCM-NB, GLCM-Discrete transform, GLCM-SVM, GLCM-DL, DL-INV3, and DL-INV2 systems obtained low $prec_{n}$ values which were 0.6240, 0.8216, 0.8356, 0.8732, 0.8689 0.8757, and 0.8170, respectively. Additionally, in terms of $reca_{l}$, the proposed IBESSDL-BCHI system obtained a maximum $reca_{l}$ value of 0.9809, whereas the GLCM-KNN, GLCM-NB, GLCM-Discrete transform, GLCM-SVM, GLCM-DL, DL-INV3, and DL-INV2 techniques attained low $reca_{l}$ values which were 0.8360, 0.8345, 0.8166, 0.8761, 0.8024 0.8707, and 0.8144, respectively.

Eventually, with regard to $F_{score}$, the proposed IBESSDL-BCHI methodology, it gained a superior $F_{score}$ value of 0.9818, whereas the GLCM-KNN, GLCM-NB, GLCM-Discrete transform, GLCM-SVM, GLCM-DL, DL-INV3, and DL-INV2 models attained low $F_{score}$ values which were 0.8222, 0.8697, 0.8469, 0.8162, 0.8792 0.8186, and 0.8642, respectively. From the detailed discussion about the results, it is evident that the proposed IBESSDL-BCHI technique yielded an effective breast cancer classification performance.

## 5. Conclusions

In this study, a new IBESSDL-BCHI method has been developed for both the recognition and classification of BC using HIs. The presented IBESSDL-BCHI model follows a series of processes, namely, MF-based noise removal, SDL feature extraction, IBES-based hyperparameter optimization, and LSTM classification. The design of the IBES algorithm aids in the precise categorization of the HIs into two major classes namely, benign and malignant. The performance of the proposed IBESSDL-BCHI mechanism was validated using a benchmark dataset, and the IBESSDL-BCHI model achieved a better general efficiency score for BC classification. Therefore, the presented model can be utilized for BC diagnosis over other models. In the future, the performance of the presented IBESSDL-BCHI algorithm can be enhanced by using an ensemble of DL models. In addition, the proposed model can also be tested on large scale real-time datasets to assure its robustness and scalability. Moreover, the computational complexity of the proposed model can be investigated in future.

## Figures and Tables

**Table 1:** Dataset details.

Total Number of Images = 1820		
Class Names	Labels	No. of Images
**Benign**		
Adenosis	A	106
Fibroadenoma	F	237
Phyllodes Tumor	PT	115
Tubular Adenona	TA	130
**Total**		**588**
**Malignant**		
Carcinoma	DC	788
Lobular Carcinoma	LC	137
Mucinous Carcinoma	MC	169
Papillary Carcinoma	PC	138
**Total**		**1232**

**Table 2:** Analytical results of the IBESSDL-BCHI approach with distinct measures and runs.

Labels	$Accu_{y},$	$Prec_{n}$	$Reca_{l}$	$Spec_{y}$	$F_{score}$	$G_{mean}$
**Run 1**						
A	98.02	80.70	86.79	98.72	83.64	92.56
F	98.96	93.95	98.31	99.05	96.08	98.68
PT	98.57	83.97	95.65	98.77	89.43	97.20
TA	99.56	96.92	96.92	99.76	96.92	98.33
DC	97.97	97.47	97.84	98.06	97.66	97.95
LC	98.19	95.61	79.56	99.70	86.85	89.06
MC	99.34	95.38	97.63	99.52	96.49	98.57
PC	98.74	98.32	84.78	99.88	91.05	92.02
**Average**	**98.67**	**92.79**	**92.19**	**99.18**	**92.27**	**95.55**
**Run 2**						
A	99.56	99.00	93.40	99.94	96.12	96.61
F	98.63	98.62	90.72	99.81	94.51	95.16
PT	99.78	96.64	100.00	99.77	98.29	99.88
TA	100.00	100.00	100.00	100.00	100.00	100.00
DC	99.56	99.12	99.87	99.32	99.49	99.60
LC	99.18	91.22	98.54	99.23	94.74	98.88
MC	99.84	98.82	99.41	99.88	99.12	99.64
PC	99.29	94.33	96.38	99.52	95.34	97.94
**Average**	**99.48**	**97.22**	**97.29**	**99.68**	**97.20**	**98.46**
**Run 3**						
A	99.67	98.08	96.23	99.88	97.14	98.04
F	99.62	99.15	97.89	99.87	98.51	98.88
PT	99.62	95.00	99.13	99.65	97.02	99.39
TA	99.73	97.71	98.46	99.82	98.08	99.14
DC	99.07	98.61	99.24	98.93	98.92	99.09
LC	99.89	99.27	99.27	99.94	99.27	99.60
MC	99.89	100.00	98.82	100.00	99.40	99.41
PC	99.56	98.51	95.65	99.88	97.06	97.74
**Average**	**99.63**	**98.29**	**98.09**	**99.75**	**98.18**	**98.91**
**Run 4**						
A	98.57	85.09	91.51	99.01	88.18	95.18
F	98.46	95.63	92.41	99.37	93.99	95.82
PT	99.01	89.43	95.65	99.24	92.44	97.43
TA	99.40	93.43	98.46	99.47	95.88	98.96
DC	97.80	98.07	96.83	98.55	97.45	97.68
LC	98.08	84.46	91.24	98.63	87.72	94.87
MC	99.62	99.39	96.45	99.94	97.90	98.18
PC	99.18	98.43	90.58	99.88	94.34	95.12
**Average**	98.76	92.99	94.14	99.26	93.49	96.66
**Run 5**						
A	99.18	93.33	92.45	99.59	92.89	95.96
F	99.51	98.31	97.89	99.75	98.10	98.81
PT	98.52	83.33	95.65	98.71	89.07	97.17
TA	99.23	92.03	97.69	99.35	94.78	98.52
DC	98.35	99.74	96.45	99.81	98.06	98.11
LC	99.34	93.10	98.54	99.41	95.74	98.97
MC	99.45	98.18	95.86	99.82	97.01	97.82
PC	99.40	96.35	95.65	99.70	96.00	97.66
**Average**	**99.12**	**94.30**	**96.27**	**99.52**	**95.21**	**97.88**

**Table 3:** Comparative analysis outcomes of the IBESSDL-BCHI approach and other existing approaches using different measures [14,24].

Methods	$Accu_{y},$	$Prec_{n}$	$Reca_{l}$	$F_{score}$
GLCM-KNN Model	0.7617	0.6240	0.8360	0.8222
GLCM-NB Model	0.7845	0.8216	0.8345	0.8697
GLCM-Discrete transform	0.8500	0.8356	0.8166	0.8469
GLCM-SVM Model	0.8500	0.8732	0.8761	0.8162
GLCM-DL Model	0.9244	0.8689	0.8024	0.8792
Deep Learning-INV3	0.9471	0.8757	0.8707	0.8186
Deep Learning-IRV2	0.8812	0.8170	0.8144	0.8642
IBESSDL-BCHI	0.9963	0.9829	0.9809	0.9818

## Data Availability

Data sharing is not applicable to this article as no datasets were generated during the current study.
